# Staying Alive; Addressing the Educational Gaps Between the See One, Do One Bag Mask Ventilation Teaching with Simulation

**DOI:** 10.1007/s40670-026-02889-z

**Published:** 2026-09-26

**Authors:** Jaclyn B Wiggins, Nicole Lock, Sarah Miller, Peter Murray

**Affiliations:** 1 Division of Neonatology, University of Virginia, Charlottesville, VA USA; 2 Division of Neonatology, University of Maryland, Baltimore, MD USA

**Keywords:** Just in Time Training, Medical Student Simulation, Low Fidelity Simulation, Bag Mask Ventilation

## Abstract

**Introduction:**

Medical students receive limited exposure to pediatric airway management during clinical clerkships, creating a critical gap in a life-saving skill. Bag-mask ventilation (BMV) is fundamental to neonatal resuscitation, yet no standardized just-in-time simulation curriculum exists for teaching this skill to medical students using low-fidelity task trainers.

**Methods:**

We developed and implemented a just-in-time, simulation-based curriculum for third-year medical students during their Surgery, Anesthesia, Obstetrics, and Gynecology clerkship block. During one-hour sessions, through the lens of Kirkpatrick’s evaluation model we evaluated level 2 (learning) by individually assessing students on BMV technique using the Helping Babies Breathe (HBB) Checklist, 2nd Edition, before and after rapid cycle deliberate practice coaching on a low-fidelity pediatric airway task trainer. We compared pre- and post-intervention scores using a paired two-tailed t-test.

**Results:**

Over one academic year, 120 medical students completed the curriculum across two sites with a 100% participation rate. Mean HBB Checklist score improved significantly from 8.3 ± 2.17 to 12.9 ± 1.22 (mean difference 4.52, 95% CI 4.09–4.95, *p* < 0.0001). Step-level analysis revealed the greatest deficits in corrective resuscitation measures at baseline, with the greatest gains observed in these steps following training. An incidental finding identified that 44% of students demonstrated incorrect hand placement over the submental space, a technique associated with airway occlusion in neonates.

**Discussion:**

This low-fidelity, just-in-time simulation curriculum significantly improved medical student BMV skills and is feasible to implement at a low cost outside of a formal simulation center.

## Introduction

Historically, medical education has taken a very linear path. Pre-clinical lectures transitioned into the “see one, do one, teach one” mindset of the clinical years [1]. With the introduction of the culture of safety in the past few decades, medical education has had to change to fill in the educational gap between “see one” and “do one.” This is where simulation-based medical education (SBME) has flourished and allowed students a safe space for rapid cycle deliberate practice and just-in-time training to hone their skills prior to their first patient interactions [2, 3].

Simulation modalities include task trainers, low- and high-fidelity mannequins, screen-based virtual reality, animal and cadaveric models, and standardized patients [1]. High-fidelity simulation is often considered the ideal teaching tool, but it is costly and typically requires a simulation laboratory with limited scheduling availability, constraining opportunities for rapid cycle deliberate practice and just-in-time training. In contrast, low-fidelity task trainers - plastic models that reproduce a single anatomical region, such as simulated skin for suturing or an airway for bag-mask ventilation - can be used almost anywhere, increasing learner exposure and repeated practice at low cost [4, 5]. Their narrow focus lets learners isolate and master one skill at a time before integrating it into clinical care, which is especially valuable for high-stakes, low-frequency procedures such as neonatal bag-mask ventilation that trainees may rarely encounter during a rotation [6, 7, 8].

In addition to Ericsson’s deliberate practice, our curriculum was structured around Kolb’s theory of experiential learning [9, 10], in which learning proceeds through four stages: concrete experience, reflective observation, abstract conceptualization, and active experimentation [11, 12, 13]. We deliberately mapped each stage onto a design element of the session: students first performed BMV on the task trainer during the initial one-on-one assessment (concrete experience); received immediate, structured feedback against the HBB Checklist during rapid cycle deliberate practice coaching (reflective observation); connected that feedback to the underlying resuscitation principles through the NRP/MR. SOPA didactic (abstract conceptualization); and then re-attempted the skill on the trainer during the post-assessment and subsequent practice (active experimentation). Structuring the workshop in this way ensured that each component reinforced a distinct stage of the experiential learning cycle. Our study aimed to measure whether the implementation of a just-in-time training session on bag-mask ventilation was an effective way to teach medical students this life-saving skill they are not often exposed to in medical school clerkships.

### Methods

We designed this study as a prospective, single-center, educational intervention study. We recruited third-year medical students from our institution to participate during their Surgery, Anesthesia, Obstetrics and Gynecology (SAGO) block of clerkship. The SAGO block spans 13 weeks, divided into 6 weeks of Surgery, 1 week of Anesthesia, and 6 weeks of Obstetrics and Gynecology. The one-hour just-in-time bag-mask ventilation workshop was delivered during the week prior to the clerkship block starting. All students attended the educational session, however participation in the study was optional. None of the 120 students declined to be in the study. Our institutional IRB approved this study. This intervention was evaluated through the lens of Kirkpatrick’s evaluation model at level 2 (learning).

We identified the need for this educational activity following the removal of the neonatal intensive care unit (NICU) rotation from the third-year medical student pediatric clerkship, which eliminated a key opportunity for students to gain exposure to neonatal airway management. In response, a team of neonatology fellows and attending faculty with expertise in neonatal resuscitation, simulation-based education, and medical student teaching convened to address this gap. We successfully obtained grant funding from the Center for Excellence in Education at the University of Virginia to support curriculum development and implementation. To ensure a rigorous and structured approach, the team participated in dedicated curriculum development educational sessions and applied Kern’s six-step approach to curriculum design, which encompasses problem identification and general needs assessment, targeted needs assessment, goals and objectives, educational strategies, implementation, and evaluation and feedback (12). Using the identified gap alongside existing evidence supporting just-in-time simulation training and low-fidelity task trainers as effective educational modalities, the team designed a one-hour workshop integrating rapid cycle deliberate practice with the HBB Checklist, 2nd Edition, as the primary assessment tool. The educational objectives, session structure, and evaluation methodology were developed collaboratively by the faculty and fellow team across iterative design sessions prior to implementation.

We completed four one-hour educational intervention sessions over a one-year time period. Using low-fidelity task trainers for simulation, we used the Helping Babies Breathe (HBB) Checklist, 2nd Edition, to assess the student’s initial technique of bag-mask ventilation and their final technique after bag-mask ventilation rapid cycle deliberate practice coaching (5). The HBB Checklist, 2nd Edition, is published through the American Academy of Pediatrics. The HBB program teaches the initial steps of neonatal resuscitation for low-resource settings. The HBB Checklist, 2nd Edition, is scored out of 14 points. The steps include placing the baby on the area for ventilation, stand at the baby’s head, check that the mask is the correct size, position the head slightly extended, apply the mask to the face, make a tight seal between the mask and the face, squeeze the bag to produce gentle movement of the chest, ventilate to produce gentle movement of the chest with each ventilation breath, ventilate at 40 breaths per minute, reapply mask, reposition head, clear mouth and nose of secretions, open the mouth, and squeeze the bag harder. During one-hour sessions, we performed an initial one-on-one evaluation of each medical student’s ability to perform bag-mask ventilation during a simulated newborn resuscitation. Concurrently, another member of the research team conducted a teaching session on intubation and advanced airway management. This didactic followed the respiratory support steps outlined in the Neonatal Resuscitation Program (NRP) guidelines. Adjust mask seal, Reposition, Suction, Open the mouth/Oxygen, increase Pressure, and Advanced airway (MR. SOPA). This was followed by instruction on how to place an advanced airway in a neonate properly with a direct laryngoscope and video laryngoscopy. Once each student had had their initial assessment and completed the didactic session, we again tested each student one-on-one on their acquisition of skills. For the simulation, students used pediatric airway trainer models and self-inflating bags with masks that fit the trainer. In total, the initial assessment was 5 min per student, the didactic took 30 min, and we ended with a post assessment that took 2–3 min per student. The same simulated newborn resuscitation scenario, task trainer, and HBB Checklist, 2nd Edition, were used for both the pre- and post-assessment, and the post-assessment was administered immediately at the end of the same one-hour session; the change in score therefore reflects immediate within-session skill acquisition on an identical task rather than a change in scenario difficulty. All assessments were performed by neonatology fellows and attending faculty with expertise in neonatal resuscitation, and the HBB Checklist, 2nd Edition, was used as a shared item-level scoring reference to standardize assessment across evaluators. No additional training beyond NRP certification was done by the assessors.

We performed all statistical analyses using GraphPad Prism. We compared pre- and post-intervention HBB Checklist scores using a paired two-tailed t-test, as each student served as their own control. We reported results as means with standard deviations. A p-value of < 0.05 was considered statistically significant, and we reported 95% confidence intervals where applicable. In addition to the paired t-test, we calculated a standardized effect size for the within-student change in total HBB Checklist score. All enrolled participants completed both assessments, yielding a complete dataset with no missing data. This outcome addressed educational objectives 1 and 2. We reported the frequency of incorrect hand placement over the submental space, an incidental observational finding noted during the educational sessions, descriptively as a proportion of the total cohort. The primary outcome was the within-student change in total HBB Checklist score following the just-in-time training intervention. We assessed step-level performance in secondary analyses across all resuscitation tasks, characterizing which specific skills demonstrated the greatest deficits at baseline and the greatest gains following instruction. To assess educational objectives 3 and 4 we have monitored the educational session since 2022 and monitored sustainability within the curriculum.

#### Results

**Fig. 1:**
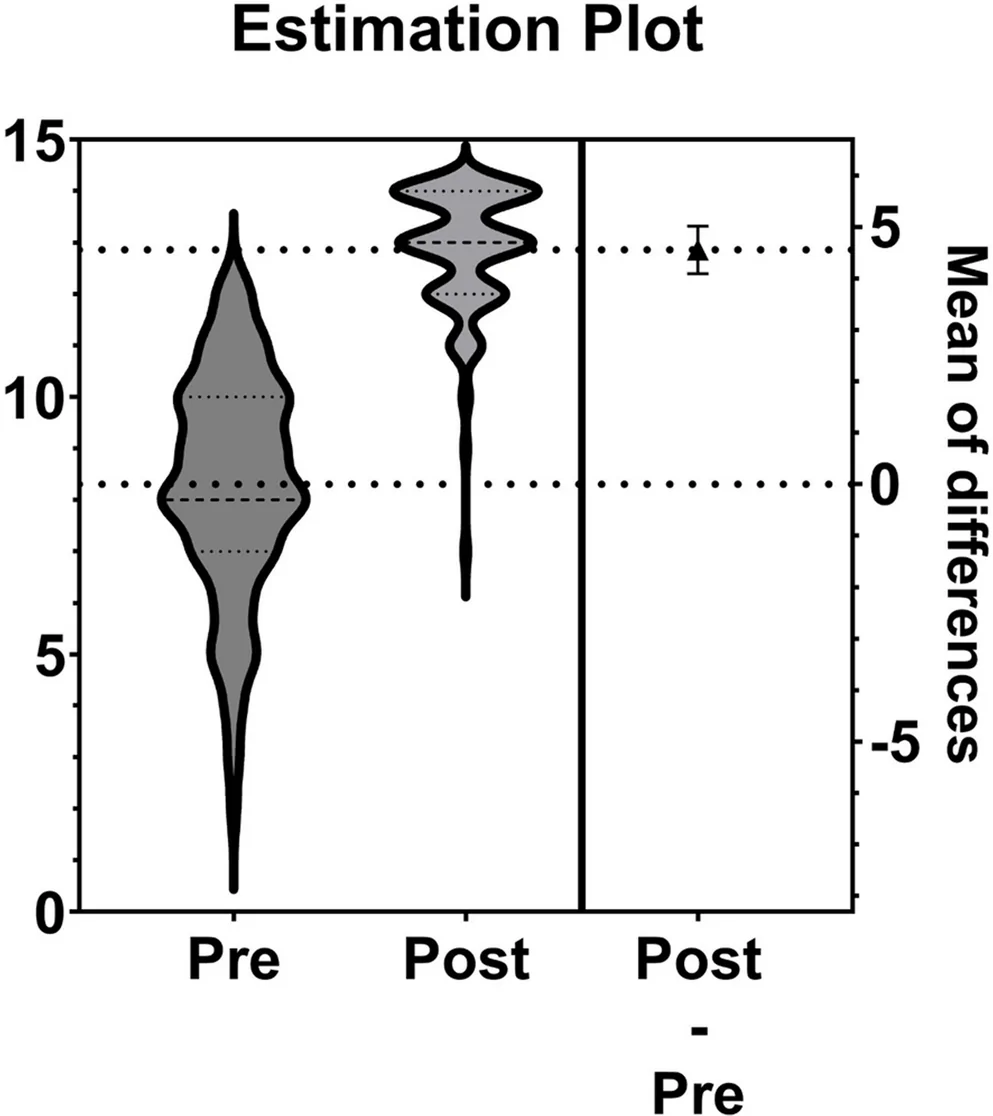
Estimation plot of the pre-test skills checklist completion and the post-test skills checklist completion

We enrolled 120 third-year medical students who completed this study from January 2022 through October 2022 at our main campus. Mean pre-intervention HBB Checklist score was 8.3 ± [2.17] and mean post-intervention score was 12.9 ± [1.22], representing a mean improvement of 4.55 points (95% CI 4.09–5.01, *p* < 0.0001) (Fig. 1). This corresponded to a large effect size (Cohen’s d_z_ ≈ 1.8). All students, except for one, improved in their scores after teaching.

**Fig. 2:**
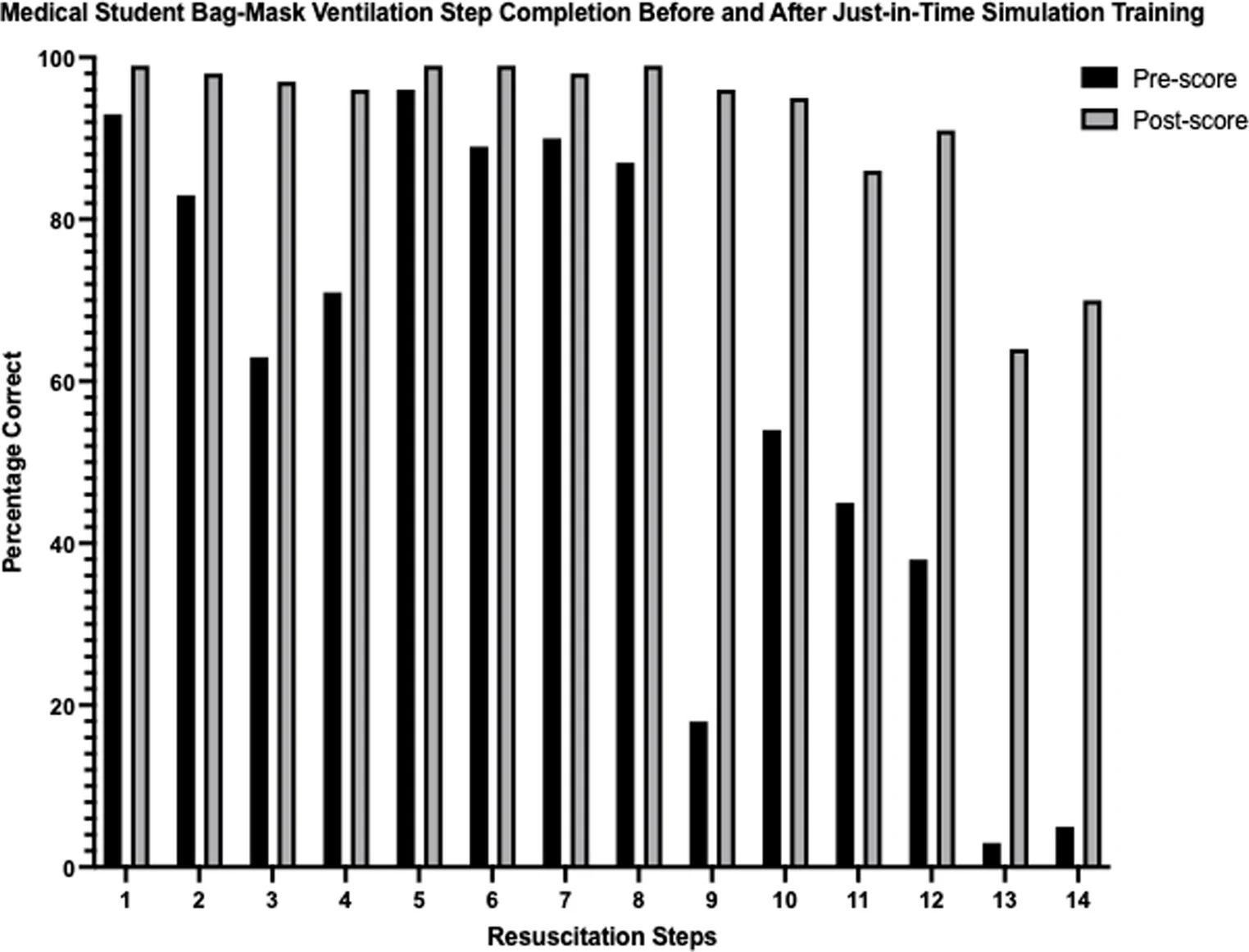
Percentage of medical students correctly performing individual neonatal resuscitation steps before and after just-in-time simulation training. Each pair of bars represents one step from the HBB Checklist, 2nd Edition, with light bars indicating pre-intervention performance and dark bars indicating post-intervention performance. Improvement across all 14 steps is demonstrated following the training intervention, with the greatest gains observed in corrective resuscitation measures

**Fig. 3:**
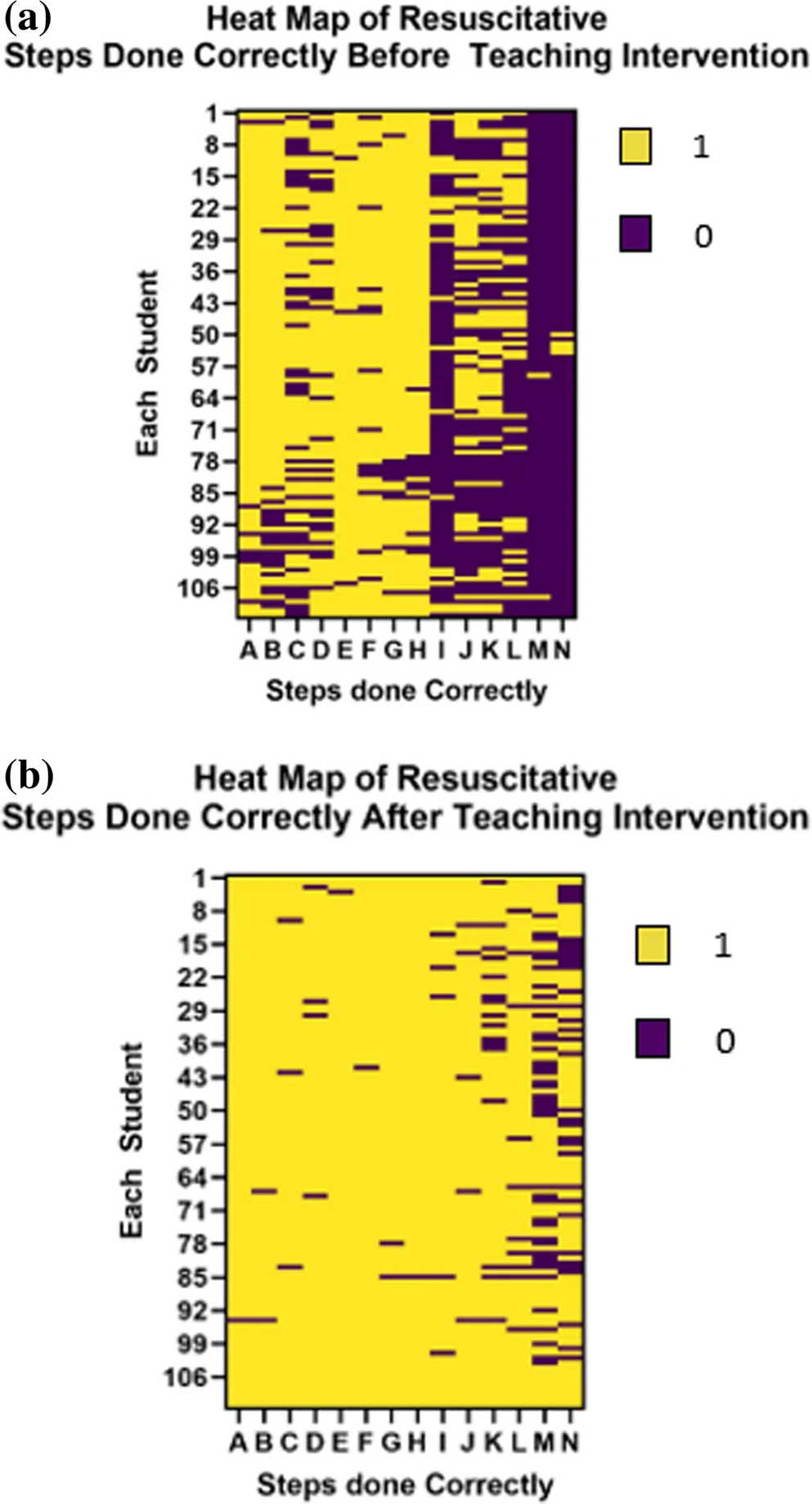
**a** Heat map of resuscitative steps done correctly before teaching per student. Each row represents an individual student and each column represents one of the 14 checklist steps. Cells are shown in two colors reflecting the binary outcome for each step: yellow indicates the step was performed correctly and purple indicates it was performed incorrectly or omitted. **b **Heat map of resuscitative steps done correctly after teaching per student. The panels show the shift from a predominance of incorrect steps at baseline (3**a**) to predominantly correct steps after training (3**b**); the pattern within each column shows which specific steps improved most, and the variation across rows shows the variability in baseline skill among students

Step-level performance analysis revealed that students were significantly more likely to correctly complete preparatory resuscitation steps (Steps 1–8) at baseline compared to corrective resuscitation steps. Following just-in-time training, performance improved across both categories, with the greatest gains observed in the corrective measures (Fig. 2). We further visualized individual student performance patterns using heat maps (Fig. 3a and b), which display step-level accuracy for each student before and after training. The heat maps demonstrate a clear shift toward correct performance across the cohort following the intervention, while also highlighting the variability in baseline skill levels among individual students.

An incidental observational finding noted during the educational sessions identified that 44% of students placed their fingers over the submental space during bag-mask ventilation, a hand positioning that may risk airway occlusion in neonates. Since study completion in 2022, the session has occurred three times per year at our main campus and satellite campus with integration of an additional pediatric component to complement the neonatal didactic.

##### **Discussion**

**Summary of Findings and Connection to the Educational Gap**

We designed this curriculum in direct response to the removal of the NICU rotation from the third-year medical student pediatric clerkship, which eliminated a key opportunity for students to gain hands-on exposure to neonatal airway management. Our findings demonstrated that a structured, low-fidelity, just-in-time simulation workshop significantly improved medical student BMV technique, with mean HBB Checklist scores improving from 8.3 to 12.9 following a single one-hour session. These results address the educational gap we identified and are consistent with prior literature demonstrating the effectiveness of just-in-time simulation for procedural skill acquisition. Our study extends this work by demonstrating that similar benefits can be achieved in medical students using an even simpler and more resource-accessible format, a low-fidelity task trainer and a freely available assessment checklist, without requiring a formal simulation center or high-fidelity equipment. The greatest gains occurred in the corrective resuscitation steps, which was expected given students’ limited prior exposure to pediatric-specific airway management. This finding underscores that the gap is not simply one of practice, but of targeted, pediatric-specific instruction that explicitly addresses the differences between adult and neonatal airways.

**Reflections on Design, Implementation, and Lessons Learned**

Several implementation insights emerged from this project that we believe will be valuable for others seeking to replicate this curriculum (Table 1). First, the one-on-one assessment model was essential to the educational impact but also the most logistically demanding component. Running simultaneous assessment and teaching stations with a small faculty and fellow team required careful scheduling and advance coordination across three sessions per year. Programs without dedicated neonatology fellows or faculty with neonatal resuscitation expertise may need to adapt this model, for example, by training senior residents or advanced practice providers as evaluators, or by running group coaching sessions as a feasible alternative to individual assessment.

**Table 1:** A step-by-step framework for designing low-fidelity, just-in-time simulation activities, aligned with Kern’s six-step approach to curriculum development

Design step (Kern’ssix-step alignment)	Design considerations and actions	Example from this bag-mask ventilation curriculum
Step 1. Problem identification & general needs assessment	Define the specific skill gap and why current training is insufficient. Confirm the skill is important (often high-stakes) yet under-taught in the existing curriculum.	*Removal of the NICU rotation from the third-year pediatric clerkship eliminated hands-on exposure to neonatal airway management*,* leaving a gap in bag-mask ventilation (BMV) skill.*
Step 2. Targeted needs assessment	Characterize the learners and verify the skill is well-suited to low-fidelity, just-in-time simulation: discrete, procedural, infrequently encountered in routine training, and assessable through observable steps.	*Third-year medical students with little neonatal resuscitation experience; BMV is a discrete psychomotor skill that can be divided into observable checklist steps.*
Step 3. Goals & objectives	Write measurable learning objectives and choose the outcome level to assess (e.g., Kirkpatrick Level 2, learning).	*By session end*,* students can perform each step of effective neonatal BMV; success measured as improvement on the HBB Checklist*,* 2nd Edition (Kirkpatrick Level 2).*
Step 4. Educational strategies	Select an inexpensive, portable task trainer and a freely available standardized checklist that doubles as the coaching guide. Structure the session around an experiential-learning cycle (assess → coach → didactic → re-attempt) using rapid cycle deliberate practice.	*Low-fidelity pediatric airway trainer with self-inflating bag and mask; HBB Checklist used for both scoring and coaching; one-on-one baseline assessment*,* RCDP coaching*,* NRP/MR. SOPA didactic*,* then re-assessment.*
Step 5. Implementation	Embed the session within a relevant clerkship block. Run parallel assessment and teaching stations to use limited faculty efficiently; plan scheduling, space, and equipment in advance.	*One-hour session during the SAGO clerkship block; simultaneous BMV assessment and intubation/advanced-airway didactic stations run by a small neonatology faculty and fellow team.*
Step 6. Evaluation & feedback	Compare pre/post performance with an appropriate paired analysis and report an effect size. Calibrate raters, track additional error patterns, and monitor sustainability and integration over time.	*Paired pre/post HBB scores analyzed with a paired t-test and effect size; the session has been sustained three times per year across two campuses since 2022.*

*Abbreviations: BMV *Bag-mask ventilation, *HBB *Helping Babies Breathe, *NICU *Neonatal intensive care unit, *NRP *Neonatal Resuscitation Program, *RCDP *Rapid cycle deliberate practice, *SAGO *Surgery, Anesthesia, Obstetrics and Gynecology

Second, the use of the HBB Checklist, 2nd Edition, as both the assessment tool and the coaching guide was highly practical and helped standardize the feedback provided across evaluators. We found that having a shared reference tool kept coaching focused and reduced variability between faculty assessors, which we had not explicitly planned for at the outset. We recommend that future implementers conduct a brief faculty calibration session prior to the first workshop to ensure inter-rater consistency.

Third, and perhaps most importantly, the incidental observation that 44% of students placed their fingers over the submental space during BMV was one of the most clinically significant findings of this project, yet it emerged informally rather than through a pre-specified assessment item. We were not equipped to quantify this finding systematically at the time of observation, and we did not initially track it as a discrete data point. In retrospect, we recommend that future implementers add hand placement as an explicit checklist item from the outset, particularly given that this error pattern appeared to stem directly from adult BMV training, highlighting a specific and correctable transfer of learning error. This finding also reinforced the value of in-person, one-on-one assessment over self report or video review for detecting subtle technique errors that may not be immediately apparent.

Finally, the timing of the workshop within the Surgery, Anesthesia, Obstetrics, and Gynecology clerkship block proved to be well-suited for implementation, as students were already engaged in a high-stakes clinical rotation and were motivated to acquire procedural competency. We found strong student engagement and a 100% voluntary participation rate, which we attribute in part to the relevance of the skill to their immediate clinical context.

**Limitations**

Several limitations should be considered when interpreting our findings. This was a single center study conducted at an academic medical center with access to neonatology fellows and faculty with simulation training experience, which may limit generalizability to community based programs or institutions without subspecialty neonatal expertise. The one-on-one format, while educationally effective, requires a favorable faculty-to-student ratio and may not be scalable at larger institutions without modification. Programs implementing this curriculum should consider how faculty time, trainer availability, and scheduling constraints at their own institutions may require adaptation of the session format. Because the same faculty both delivered the instruction and scored the assessments, the potential for assessor bias cannot be excluded; independent or blinded evaluation would strengthen future studies.

We did not assess skill retention beyond the immediate post-intervention period [13]. The durability of the gains we observed remains unknown, and this represents an important gap in our evaluation. Because the checklist uses a finite 14-point scale and the post-assessment was administered immediately after coaching, the near-maximal post-intervention scores likely reflect a ceiling effect. This constrains our ability to detect further gains at the upper end of the scale and, together with the absence of a delayed reassessment, limits interpretation of longer-term retention. Future iterations of this curriculum should include a follow-up assessment at the end of the clerkship block or at the start of the subsequent clinical year to determine whether a booster session is needed to maintain competency.

The HBB Checklist, 2nd Edition, was not validated specifically for use with medical student learners in a simulation based setting; it was developed for low resource birth attendant training. While it provided a practical and standardized framework, future evaluators should consider pairing it with a validated pediatric BMV assessment tool or a confidence survey to capture both objective performance and self-efficacy outcomes. We did not survey students on their confidence before and after training, which limited our ability to assess whether the session impacted perceived self-efficacy in addition to observed skill. Additionally, while all 120 students chose to participate despite optional enrollment, this near perfect voluntary participation rate may reflect social desirability or selection factors related to a closely supervised clerkship context, and should not be assumed to replicate across all institutional settings.

**Future Directions, Implications and Suitability Criteria**

This curriculum is designed to be adapted and implemented broadly. The materials provided in this publication — including the session structure, assessment guide, and facilitation notes — require only a low-fidelity pediatric airway trainer and self-inflating bag, both of which are widely available at low cost. Institutions without neonatology programs could implement this workshop using pediatric emergency medicine faculty, anesthesiology instructors, or other providers with neonatal resuscitation expertise. The curriculum could also be readily adapted for other learner groups, including physician assistant and nurse practitioner students, nursing students, and first-year residents beginning NICU or pediatric rotations.

Future refinements to this curriculum should focus on three priorities. First, adding a brief validated confidence survey before and after the session would allow programs to assess whether skill gains translate to improved self-efficacy, which is relevant to how students approach real clinical encounters. Second, incorporating a structured booster session at two to three months post-training could address skill decay and reinforce the most commonly missed corrective steps. Third, explicitly adding hand placement as a scored checklist item — and building a brief instructional segment on the anatomical differences between adult and pediatric airways — would directly address the transfer of learning error we observed and strengthen the pedagogical intentionality of the curriculum. This approach is best suited to educational gaps that share several features: the target skill is discrete and procedural rather than diffuse or knowledge-based; it can be practiced on an inexpensive, portable task trainer without a formal simulation center; performance can be captured with a standardized, observable checklist; and the skill is high-stakes but infrequently encountered in routine training, so that structured deliberate practice meaningfully offsets limited real-world exposure. Conversely, gaps that require high-fidelity physiologic feedback, team-based crisis management, or longitudinal patient relationships are less likely to be addressed adequately by this low-fidelity, single-session model [14]. Programs deciding whether to adopt this approach when time and resources are limited can use these criteria to judge fit before investing in curriculum development.

More broadly, our findings contribute to a growing body of evidence supporting the integration of pediatric-specific procedural simulation into medical student clerkship curricula. As pediatric clinical exposure for medical students continues to shrink due to competing curricular demands and the protected status of children as a vulnerable research population, structured simulation-based alternatives become increasingly important for ensuring baseline procedural competency before graduation. We believe this curriculum offers a replicable, low-cost, and evidence-based model that can meaningfully address this gap across a wide range of training environments.

## Data Availability

The datasets generated and analyzed during the current study are available from the corresponding author on reasonable request.
